# Gene Cloning, Expression and Characterization of a Novel Xylanase from the Marine Bacterium, *Glaciecola mesophila* KMM241

**DOI:** 10.3390/md11041173

**Published:** 2013-04-08

**Authors:** Bing Guo, Ping-Yi Li, Yong-Sheng Yue, Hui-Lin Zhao, Sheng Dong, Xiao-Yan Song, Cai-Yun Sun, Wei-Xin Zhang, Xiu-Lan Chen, Xi-Ying Zhang, Bai-Cheng Zhou, Yu-Zhong Zhang

**Affiliations:** The State Key Lab of Microbial Technology, Marine Biotechnology Research Center, Shandong University, Jinan 250100, China; E-Mails: guo321bing@163.com (B.G.); lipingyipeace@163.com (P.-Y.L.); yueyongsheng1124@163.com (Y.-S.Y.); zhaohuilin1984@yahoo.cn (H.-L.Z.); Ds518@126.com (S.D.); xysong@sdu.edu.cn (X.-Y.S.); suncy@sdu.edu.cn (C.-Y.S.); zhangweixin1984@163.com (W.-X.Z.); cxl0423@sdu.edu.cn (X.-L.C.); zhangxiying@sdu.edu.cn (X.-Y.Z.); bczhou@ms.qdio.ac.cn (B.-C.Z.); zhangyz@sdu.edu.cn (Y.-Z.Z.)

**Keywords:** xylanase, XynB, cold-active, *Glaciecola mesophila* KMM241, carbohydrate-binding module

## Abstract

Marine xylanases are rather less studied compared to terrestrial xylanases. In this study, a new xylanase gene, *xynB*, was cloned from the marine bacterium, *Glaciecola mesophila* KMM241, and expressed in *Escherichia coli*. *xynB* encodes a multi-domain xylanase XynB of glycoside hydrolase (GH) family 8. The recombinant XynB comprises an *N*-terminal domain (NTD) with unknown function and a catalytic domain, which is structurally novel among the characterized xylanases of GH family 8. XynB has the highest identity (38%) to rXyn8 among the characterized xylanases. The recombinant XynB showed maximal activity at pH 6–7 and 35 °C. It is thermolabile and salt-tolerant. XynB is an *endo*-xylanase that demands at least five sugar moieties for effective cleavage and to hydrolyze xylohexaose and xylopentaose into xylotetraose, xylotriose and xylobiose. NTD was expressed in *Escherichia coli* to analyze its function. The recombinant NTD exhibited a high binding ability to insoluble xylan and avicel and little binding ability to chitosan and chitin. Since the NTD shows no obvious homology to any known carbohydrate-binding module (CBM) sequence in public databases, XynB may contain a new type of CBM.

## 1. Introduction

Xylan, which together with lignin and cellulose constitutes the major components of plant cell walls, is the most abundant hemicellulose in nature and represents an important type of renewable resource [1]. Complete biodegradation of xylan requires synergy of a variety of xylanolytic enzymes. According to the amino acid sequences of catalytic domains, enzymes with xylanase activity can be categorized into glycoside hydrolase (GH) families 5, 7, 8, 10, 11, 16, 26, 43, 52 and 62. However, only those sequences classified into families 5, 7, 8, 10, 11 and 43 contain truly distinct catalytic domains with a demonstrated *endo*-1,4-β-xylanase activity. Family 16, 52 and 62 enzymes are bifunctional enzymes containing two catalytic domains, a family 10 or 11 xylanase domain and a glycosidase domain. Those enzymes classified into family 26 are *endo*-1,3-β-xylanases [2]. *Endo*-β-1,4-xylanases (EC 3.2.1.8) are glycosidases, which cleave the internal β-1,4-xylosidic bonds of the main chain of xylan, producing unbranched or branched *xylo*-oligosaccharide [3] and, thus, are widely used in food, paper, textile and many other industries [4,5]. Xylanases from GH families 10 and 11 have been widely investigated. In contrast, xylanases from other families are less studied. GH family 8 comprises endoxylanases, as well as xylose-releasing *exo*-oligoxylanases (EC 3.2.1.156) [6]. Until now, only four GH family 8 *endo*-xylanases have been identified, including recombinant xylanase XynY from *Bacillus* sp. KK-1 [7], xylanase PhXyl from *Pseudoalteromonas haloplanktis* [8], xylanase XYL6806 from an insect gut microbe [9] and xylanase rXyn8 from an uncultured bacterium [10]. In addition, two GH family 8 *exo*-xylanases have been reported, one from *Bacillus* *halodurans* [11] and the other from *Bifidobacterium adolescentis* [12].

At present, there is an increasing interest in searching for new sources of xylanases, especially those with extremophilic properties, as these enzymes may have an advantage when applied at extreme conditions [2]. Marine microorganisms have been regarded as a reservoir, not only for novel natural products, but also for valuable genes and enzymes. Several xylanases produced by marine microorganisms from special ecological habitats, such as the deep-sea hydrothermal field [13], the Antarctic marine soil [8] and marine sediment [14], were shown to have special properties, such as hyperthermostability [13], cold adaptation [8,14] and salt-tolerance [14,15]. However, studies on marine xylanases are still rare compared to those on terrestrial xylanases. 

*Glaciecola* *mesophila* KMM241 was isolated from a specimen of marine invertebrate *Halocynthia aurantium* [16]. In our previous studies, a cold-adapted and salt-tolerant GH family 10 *endo*-β-1,4-xylanase (XynA) from *G.* *mesophila* KMM241 was expressed and characterized [14], and the effect of XynA on bread baking was studied [17]. In this study, another xylanase, XynB, from *G. mesophila* KMM241 was expressed and characterized, which showed that XynB was a GH family 8 xylanase containing an *N*-terminal domain and a catalytic domain. Moreover, the xylan-binding function of the *N*-terminal domain was studied, which suggests that this domain may represent a new type of carbohydrate-binding module (CBM).

## 2. Materials and Methods

### 2.1. Strains, Plasmids and Chemicals

*G.* *mesophila* KMM241 was purchased from DSMZ (DSM 15026^T^). *Escherichia coli* DH5α and BL21 were used for gene cloning and expression, respectively. The plasmid, pGME-T (Novagen, Billerica, MA, USA), was used for gene cloning, and the plasmid, pET-22b(+) (Novagen, USA), was used for expression. Beech wood xylan, oat spelt xylan and *xylo*-oligosaccharides (xylose, xylobiose, xylotriose, xylotetraose, xylopentaose and xylohexaose) were all purchased from Sigma (St louis, MO, USA).

### 2.2. Gene Cloning of *xynB*

Genomic DNA of *G.* *mesophila* KMM241 was prepared with the method described by Saito and Miura [18]. Blast analysis in NCBI showed that the 16S rRNA gene sequence of *G.* *mesophila* KMM241 had more than 99% identity to that of *Pseudoalteromonas atlantica* T6c. The whole genome of strain T6c was sequenced and released in GenBank, which revealed that there is a gene encoding a hypothetical *endo*-1,4-β-xylanase in the genome. Based on the 5′ end and 3′ end sequence of this gene, two primers were designed as follows: XB22-1,5′-CGACGGATCCGGAAGTGAGCATTGATCAC-3′; XB22-2,5′-CGACGCTCGAGTTCAGGCTCGTTTTC-3′, in which the cleavage sites of restriction enzymes, *Bam*H I and *Xho* I, were added and underlined. With these primers and the genomic DNA of strain KMM241 as the template, PCR amplification was performed by Fastpfu polymerase with the following procedure: 95 °C for 5 min, 30 cycles of 94 °C for 30 s, 50 °C for 1 min and 72 °C for 2 min and 72 °C for 10 min. An about 2800 bp DNA fragment was amplified and then sequenced. Sequence analysis showed that it contains a 2739 bp ORF (open reading frame) encoding a hypothetical *endo*-1,4-β-xylanase. Then, this gene, named *xynB*, was deposited in GenBank under accession No. JF514216. 

### 2.3. Expression and Purification of XynB

An expression plasmid pET22b-*xynB* was constructed by ligating gene *xynB* into the *Bam*H I–*Xho* I restriction sites of the pET22b plasmid. Plasmid pET22b-*xynB* was introduced into *E. coli* BL21(DE3) competent cells. The transformed strain was cultured in Luria-Bertani medium supplemented with 0.1 mg/mL ampicillin overnight at 37 °C. Then, the culture was diluted 100-fold in fresh Luria-Bertani medium with 0.1 mg/mL ampicillin and incubated at 37 °C. When the culture reached an optical density of 1.0 at 600 nm, isopropyl-β-d-thiogalactopyranoside (IPTG, Sigma, USA) was added to a final concentration of 0.2 mM, and the culture was further cultivated at 15 °C for 30 h. Then, the culture was centrifuged at 10,000× *g* at 4 °C for 5 min. The cells were collected, resuspended in 50 mM phosphate buffer (pH 7.0) and sonicated. The extract was subjected onto a DEAE-Sepharose Fast Flow column (GE Healthcare, Fairfield, CT, USA) equilibrated with the same buffer. The elution was performed with a linear gradient of 0–0.8 M NaCl. The fraction with xylanase activity was collected and subjected onto a His-Bind metal chelating column (GE Healthcare, USA) equilibrated with 50 mM phosphate buffer (pH 7.4) containing 500 mM NaCl and 5 mM imidazole and eluted with 50 mM phosphate buffer (pH 7.4) containing 500 mM NaCl and 150 mM imidazole. The purity of recombinant XynB was detected by SDS-PAGE with the method of Laemmli [19].

### 2.4. Enzyme Assay and Protein Determination

To measure the xylanase activity of XynB, a reaction mixture containing 0.01 mL enzyme and 0.09 mL beech wood xylan (10 mg/mL) dissolved in 50 mM Tris-HCl buffer (pH 7.0) was incubated at 35 °C for 10 min. After incubation, 0.15 mL dinitrosalicylic acid (DNS) was added to end the reaction. The reducing sugar released in the mixture was determined, with xylose as the standard using the DNS method [20]. One unit of enzyme activity was defined as the amount of enzyme capable of releasing 1 μmol of xylose per minute under the assay conditions. Protein concentration was estimated with Bradford’s method [21].

### 2.5. Enzyme Characterization

To determine the optimal pH of XynB, xylanase activity was assayed at 35 °C in different buffers ranging from pH 4.0 to pH 11.0. The buffers used were 50 mM citrate buffer for pH 4.0–6.0, 50 mM phosphate buffer for pH 6.0–8.0, 50 mM Tris-HCl buffer for pH 8.0–9.0 and 50 mM glycine-NaOH buffer for pH 9.0–11.0. pH stability was determined by measuring the residual activity of XynB at pH 7.0 and 35 °C after the enzyme was incubated in the buffers at 15 °C for 1 h. The optimal temperature for xylanase activity was determined in 50 mM phosphate buffer (pH 7.0). The thermostability of XynB was determined by measuring the residual activity after incubation at 25 °C, 35 °C and 45 °C for different periods of time. To analyze the effect of different metal ions and chemical reagents on the activity, 15 kinds of chemicals, including SnCl_2_, CaCl_2_, KCl, SrCl_2_, MgCl_2_, FeCl_3_, CuCl_2_, LiCl_2_, MnCl_2_, NiCl_2_, ZnCl_2_ and CoCl_2_, all from Sinopharm Chemical Reagent Co., Ltd. (Shanghai, China), as well as Urea, EDTA and SDS from Sigma (USA), were added to the reaction mixture at final concentrations of 1 mM and 5 mM, respectively, and then the xylanase activity was assayed at pH 7.0 and 35 °C. To investigate the effect of NaCl on the xylanase activity of XynB, 0.1–4.0 mol/L NaCl was added to the reaction mixture, and then, the xylanase activity was assayed at pH 7.0 and 35 °C. Substrate specificity of XynB was tested by measuring its activity toward beech wood xylan, oat spelt xylan, carboxymethylcellulose, laminarin, mannan, starch and chitosan. All reactions were conducted in 50 mM phosphate buffer (pH 7.0) with a final substrate concentration of 10 mg/mL at 35 °C for 10 min. The kinetic parameters of XynB on beech wood xylan and oat spelt xylan were determined at 35 °C by Lineweaver-Burk plots, which were made by linear regression, with initial rates determined between 1.0 and 15 mg/mL of substrates.

### 2.6. Analysis of the Products of *Xylo*-Oligosaccharides Hydrolyzed by XynB

A 50 μL reaction solution containing 0.2 μg XynB in 50 mM phosphate buffer (pH 7.0) and 1 mg/mL *xylo*-oligosaccharides was incubated at 15 °C for 12 h. After incubation, the solution was boiled for 5 min and then centrifuged at 10,000× *g* for 10 min. The supernatant was filtrated through a 0.22 μm cellulose acetate membrane for HPLC analysis. The hydrolytic products were analyzed using LC-10AD high-performance liquid chromatography (Shimadzu, Japan), equipped with an HPX-42C Aminex column (Bio-Rad, Hercules, CA, USA) and an RID-10A refractive index detector (Shimadzu, Japan). The column was maintained at 75 °C and eluted with ddH_2_O at a flow rate of 0.4 mL/min. Xylose and a mixture of *xylo*-oligosaccharides (x2–x6) were used as the standard.

### 2.7. Expression and Purification of the *N*-Terminal Domain

A DNA fragment coding for the *N*-terminal domain (NTD) of XynB (Glu44-Arg584) was amplified from *G.* *mesophila* KMM241 by PCR with the primers, 5′-CGACCATATGGAAGTGAGCATTGATCAC-3′ and 5′-CGACCTCGAGGCGAATATCGTTTGAATC-3′, in which the cleavage sites of restriction enzymes, *Bam*H I and *Xho* I, were added and underlined. An expression plasmid, pET22b-*ntd*, was constructed for the expression of NTD of XynB. pET22b-*ntd* was transformed into *E. coli* BL21-(DE3) and induced by 0.5 mM IPTG at 15 °C for 30 h. The recombinant NTD was expressed as a *C*-terminal His_6_-tagged protein and purified from the cell extract with a His-Bind metal chelating column. The purity of the recombinant NTD was detected by SDS-PAGE with the methods of Laemmli [19].

### 2.8. Insoluble Polysaccharide-Binding Assay of the *N*-Terminal Domain

Insoluble xylan was prepared from oat spelt xylan by using the method of Pason *et al.* [22]. Avicel, chitin and chitosan were purchased from Sigma (USA). The binding ability of the NTD toward these four insoluble polysaccharides was determined by using the method of Valenzuela *et al.* [23]. The NTD (0.1 mg) was mixed with insoluble polysaccharides (20 mg) in 20 mM sodium phosphate buffer (pH 7.0) in a final volume of 0.2 mL, which was incubated on ice for 1 h with occasional stirring. After incubation, the mixture was centrifuged at 13,000 rpm for 10 min. The supernatant was carefully removed for SDS-PAGE analysis. Pellets were washed with 0.4 mL of the same buffer three or four times, and then, the pellets were resuspended in 0.1 mL of 10% SDS and boiled for 10 min to denature the bound protein. The proteins in the supernatant and the wash buffer of the last time, as well as the released protein from the pellets were analyzed by SDS-PAGE. Bovine serum albumin (BSA, Sigma, USA) instead of the NTD was used as a negative control. 

## 3. Results

### 3.1. Gene Cloning and Sequence Analysis

**Figure 1:**
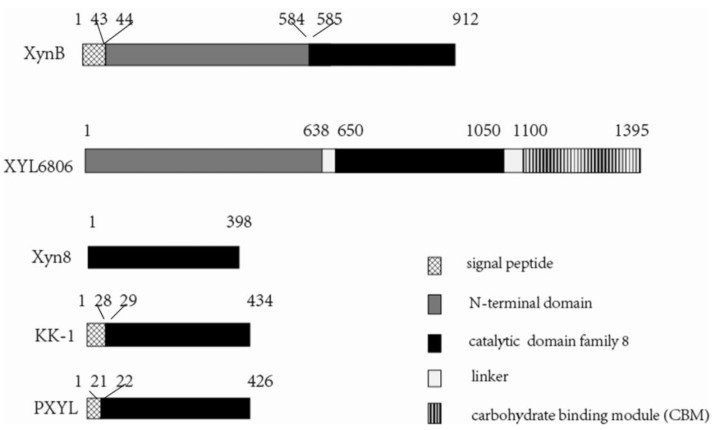
Schematic diagrams of the domain structures of XynB and four other characterized GH family 8 *endo*-xylanases. XynB, the xylanase in this study (AEC33258); X6806, a xylanase from insect guts (AAS85781) [9]; Xyn8, a cold-active xylanase enzyme from an environmental DNA library (ABB71891) [10]; KK-1, a xylanase from *Bacillus* sp. KK-1 (AAC27700) [7]; PH, a psychrophilic xylanase from *Pseudoalteromonas haloplanktis* TAH 3a (AJ427921) [8].

**Figure 2:**
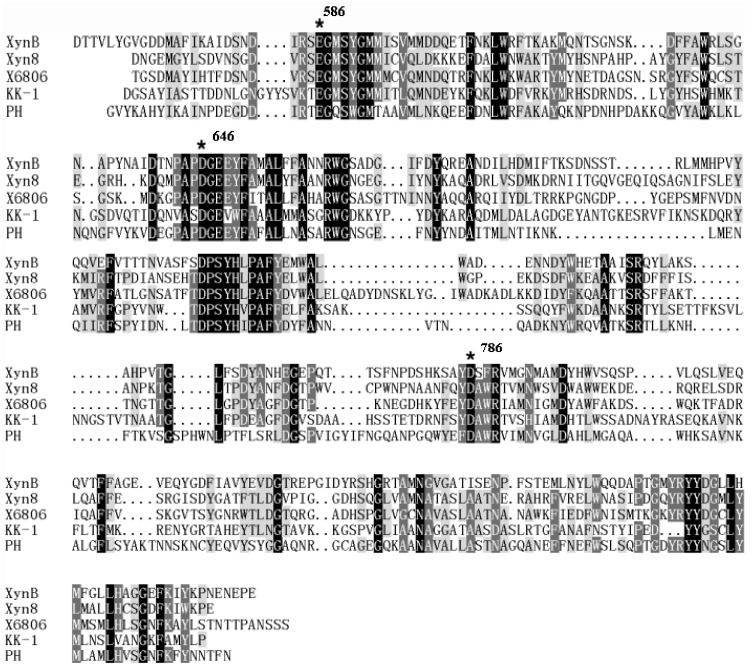
Alignment of catalytic domains of XynB and some other characterized GH family 8 xylanases. Stars above residues indicate the conserved catalytic amino acids. Identical residues are shaded in black. XynB, the xylanase in this study (AEC33258); Xyn8, a cold-active xylanase enzyme from an environmental DNA library (ABB71891) [10]; X6806, a xylanase from insect guts (AAS85781) [9]; KK-1, a xylanase from *Bacillus* sp. KK-1 (AAC27700) [7]; PH, a psychrophilic xylanase from *Pseudoalteromonas haloplanktis* TAH 3a (AJ427921) [8].

Gene *xynB* was cloned from genomic DNA of *G.* *mesophila* KMM241. The ORF comprises 2739 bp, including an ATG start codon and a TAA stop codon. It encodes a protein of 912 amino acid residues with a calculated *M_r_* of 100,502 Da, which was predicted to be a GH family 8 xylanase (named XynB). The amino acid sequence of XynB was searched against GenBank databases. Results showed that XynB is a multi-domain enzyme containing a predicted 43-residue signal peptide at the *N*-terminus (Met1-Ala43), an NTD following the signal peptide (Glu44-Arg584) and a catalytic domain at its *C*-terminus (Ser585-Glu912) (Figure 1). The catalytic domain showed extensive homology with xylanases of GH family 8 [24]. In addition, the multiple sequence alignment (see Figure 2) revealed that XynB contained glutamate and aspartate residues (Glu586, Asp646 and Asp786), which are considered to be crucial for catalytic activity of GH family 8 [10]. XynB had high amino acid sequence identities to some hypothetical xylanases in databases, such as the xylanase from *Pseudoalteromonas atlantica* T6c (98%, YP660649) and the xylanase from *Glaciecola chathamensis* S18K6 (80%, ZP11354175). In contrast, XynB had low identities to characterized xylanases. Among characterized xylanases, XynB had highest identity (38%) to the xylanase rXyn8 from an uncultured bacterium [10].

### 3.2. Expression and Characterization of XynB

**Figure 3:**
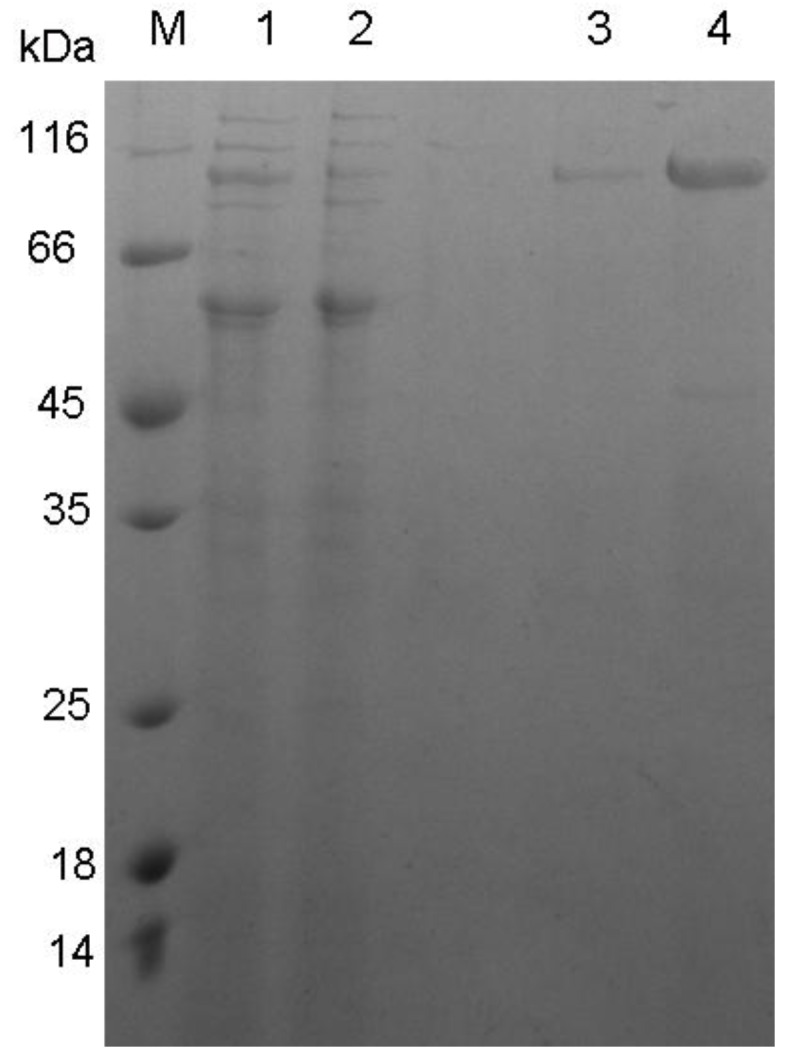
SDS-PAGE analysis of purified recombinant XynB. M, protein molecular weight marker; lanes 1 and 2, proteins in two different tubes after DEAE-Sepharose ion exchange chromatography; lanes 3 and 4, purified XynB in two different tubes after Ni-affinity chromatography.

Gene *xynB* was expressed in *E. coli*, and the recombinant XynB was purified (Figure 3). SDS-PAGE analysis indicated that the recombinant XynB had an apparent *M_r_* of about 95 kDa (Figure 3). Since the theoretical molecular mass calculated from the sequence of XynB without the signal peptide is 97.1 kDa, the mature XynB should be composed of the NTD and the catalytic domain. 

The purified XynB was then characterized. XynB had high activity on natural xylans, such as beech wood xylan and oat spelt xylan, and no detectable activity on cellulose or other glucose-formed polysaccharides, such as laminarin, mannan, chitosan and starch, which suggests that XynB is a strict xylanase. Of the two types of xylan tested, XynB showed a higher specific activity toward beech wood xylan (143 ± 1.8 U/mg protein) and a lower specific activity toward soluble oat spelt xylan (108 ± 2.3 U/mg protein) under optimal conditions (pH 7.0 and 35 °C).

**Figure 4:**
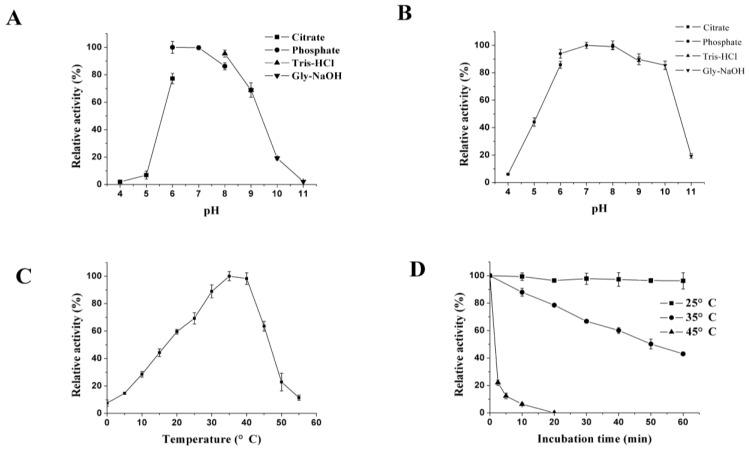
pH and temperature optimums and stabilities of XynB. (**A**) Effect of pH on XynB activity. Activities at various pH were assayed at 35 °C; (**B**) pH stability of xynB. Residual activities after incubation at various pH were assayed at pH 7.0 and 35 °C; (**C**) Effect of temperature on XynB activity. The assay was performed at pH 7.0 in phosphate buffer; (**D**) Thermostability of XynB. The enzyme was incubated at 25 °C, 35 °C and 45 °C for different periods of time, and then, the residual activity was assayed at 35 °C.

With beech wood xylan as the substrate, XynB showed the highest activity at pH 6.0–7.0 (Figure 4A). It was stable between pH 6.0 and 10.0, retaining more than 80% activity after incubation at pH 6.0–10.0 for 1 h (Figure 4B). The optimal temperature for XynB was 35 °C. It retained 7.5% and 14.6% activity at 0 °C and 5 °C, respectively, and had no detectable activity at 60 °C (Figure 4C). As shown in Figure 4D, XynB exhibited very low thermostability, retaining less than 40% activity after 60-min incubation at 35 °C and losing all the activity after 20-min incubation at 45 °C. These results indicated that XynB is thermolabile. 

**Figure 5:**
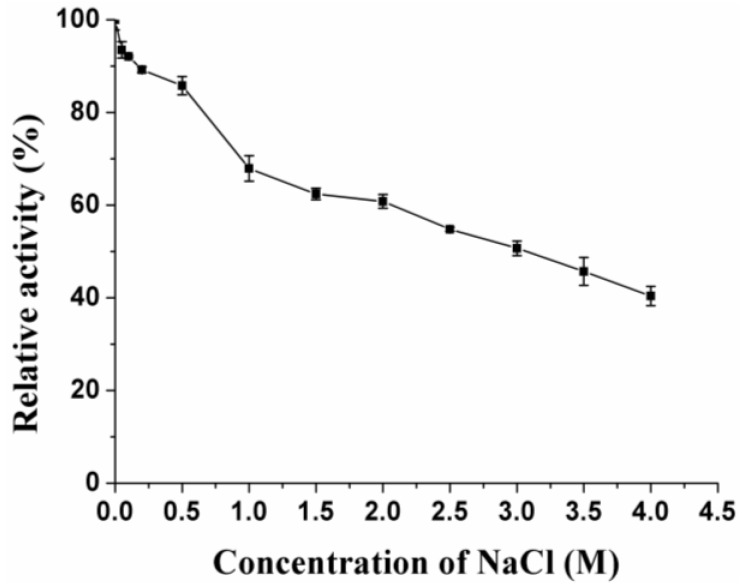
Effect of NaCl on the activity of recombinant XynB. The enzyme activity was measured at 35 °C in phosphate buffer (pH 7.0) containing 0–4.0 M NaCl.

Among all the tested metal ions, Co^2+^ (1 mM and 5 mM) had the strongest inhibitory effect on the activity, while Zn^2+^, Ni^2+^, Mn^2+^ and Li^2+^ at a concentration of 5 mM had significantly inhibitory effects. No metal ions or chemical reagents showed obvious stimulative effects on the activity (Table 1). The NaCl concentration of the seawater is about 3%. Since *G.* *mesophila* KMM241 was isolated from a marine invertebrate animal, the effect of NaCl on XynB activity was evaluated. NaCl displayed no evident activation effect on XynB activity. However, XynB showed some salt-tolerant ability, retaining approximately 40% activity in 4.0 M NaCl (Figure 5).

**Table 1:** Effect of metal ions and chemical reagents on the activity of XynB *.

Metal ions and chemical reagents	Relative activity (%)	
	1 mM	5 mM
control	100 ± 1.2	100 ± 0.5
Urea	99.2 ± 3.1	101.5 ± 2.4
Sn^2+^	96.3 ± 0.7	100.4 ± 1.7
Ca^2+^	97.9 ± 0.8	95.6 ± 1.1
K^+^	101.3 ± 2.1	93.7 ± 0.8
Sr^2+^	95.7 ± 1.3	94.5 ± 2.3
Mg^2+^	98.7 ± 0.3	89.7 ± 1.9
Fe^3+^	92.5 ± 0.5	87.7 ± 3.6
Cu^2+^	95.7 ± 1.9	86.4 ± 2.7
Li^2+^	94.0 ± 0.8	76.2 ± 0.8
EDTA	85.8 ± 2.1	67.6 ± 1.4
SDS	84.9 ± 1.0	65.0 ± 1.8
Mn^2+^	73.4 ± 2.2	54.2 ± 0.6
Ni^2+^	90.7 ± 3.8	49.7 ± 0.9
Zn^2+^	81.1 ± 0.7	39.0 ± 2.5
Co^2+^	64.2 ± 0.6	8.5 ± 2.2

* The activity of XynB in the presence of 1 mM and 5 mM of each reagent was measured at pH 7.0 and 35 °C, respectively. The activity of XynB without any reagent was taken as control (100%). Each experiment was performed in triplicate.

Kinetic parameters of XynB on beech wood xylan and oat spelt xylan were determined at 35 °C (Table 2). XynB had lower *K*_m_ and higher *k*_cat_/*K*_m_ to beech wood xylan than to oat spelt xylan, indicating that XynB had stronger affinity and higher catalytic efficiency to beech wood xylan than to oat spelt xylan.

**Table 2:** Kinetic parameters of XynB on two types of xylan *.

Xylan	*K*_m_ (mg/mL)	*V*_max_ (mmol/min·mg)	*k*_cat_ (1/s)	*k*_cat_/*K*_m_ (mL/mg·s)
Beech wood xylan	5.82	0.38	609	104.64
Oat spelt xylan	11.86	0.44	712	60.03

* The *K*_m_ and *V*_max_ values were determined by Lineweaver-Burk plots, which was made by linear regression, with initial rates determined with 1.0–15.0 g/L substrate at 35 °C using an enzyme concentration ([E]) of 36 μg/mL. *k*_cat_ values were calculated with the formula *k*_cat_ = *V*_max_/[E].

### 3.3. Hydrolysis Product Analysis

**Figure 6:**
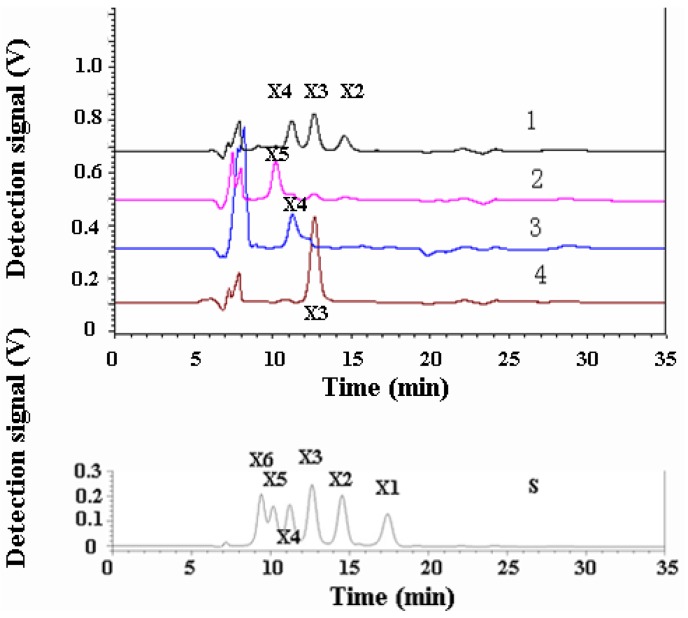
HPLC analysis of the products of *xylo*-oligosaccharides hydrolyzed by XynB. S, a mixture of x1–x6; 1, hydrolysis products of xylohexaose; 2, hydrolysis products of xylopentaose; 3, hydrolysis products of xylotetraose; 4, hydrolysis products of xylotriose.

*Xylo*-oligosaccharides, x3–x6, were hydrolyzed by XynB at 15 °C for 12 h, respectively, and the products were analyzed using HPLC. As shown in Figure 6, XynB could not hydrolyze xylotetraose and xylotriose and could hydrolyze xylohexaose and xylopentaose. Xylohexaose was completely hydrolyzed, producing xylobiose, xylotriose and xylotetraose. The hydrolysis efficiency to xylopentaose was relatively low, producing a small amount of xylotriose and xylobiose. No xylose was detected in all hydrolysis process. These results showed that XynB is a strict *endo*-β-1,4-xylanase exhibiting an action pattern with a demand of at least five sugar moieties for effective cleavage.

### 3.4. Insoluble Polysaccharide-Binding Ability of the *N*-Terminal Domain

**Figure 7:**
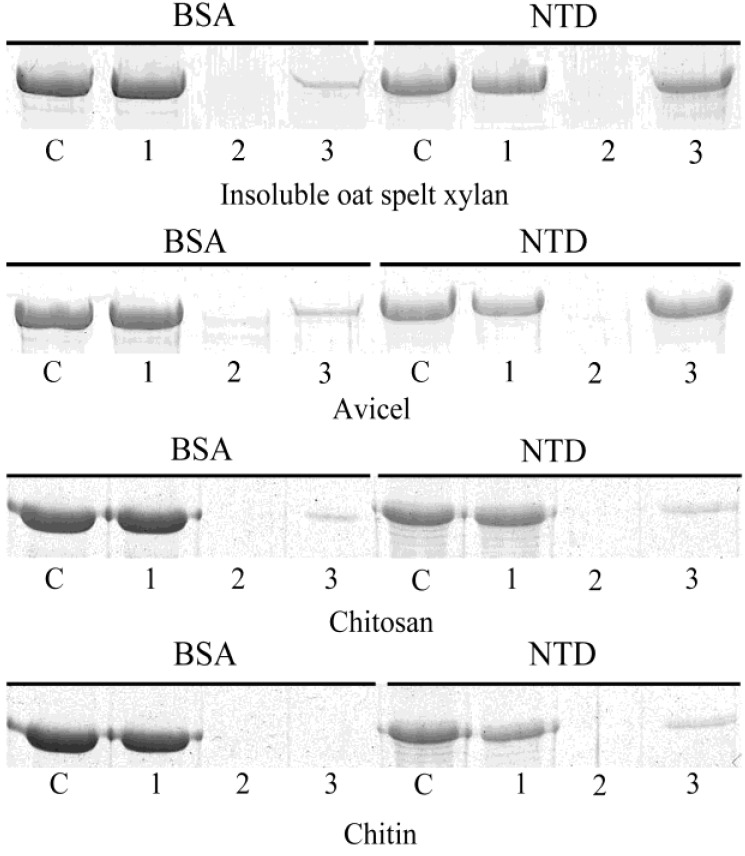
SDS-PAGE analysis of binding of the recombinant *N*-terminal domain (NTD) to different insoluble polysaccharides. Bovine serum albumin (BSA) (0.1 mg) was used as a negative control. Proteins were mixed with the insoluble polysaccharides for 1 h; bound and unbound fractions were separated by centrifugation and analyzed by 12.5% SDS-PAGE. The proteins in the gels were visualized by staining with 0.1% Coomassie blue R250. Lane C, control protein; lane 1, unbound fraction; lane 2, protein in the wash buffer; lane 3, fraction adsorbed to the polymer.

Some xylanases own one or several CBMs. In order to investigate whether the NTD of XynB functions as a CBM, we expressed NTD and examined the binding ability of the recombinant NTD to four insoluble polysaccharides by SDS-PAGE. As shown in Figure 7, the recombinant NTD exhibited strong binding ability to insoluble oat spelt xylan and avicel, but little binding ability to chitosan and chitin. This result suggested that the NTD of XynB may function as a substrate-binding domain during xylan hydrolysis. CBMs are now classified into 66 families on the basis of amino acid sequence similarities in the CAZy database. Blast analysis indicated that the NTD of XynB has no obvious homology to any CBM sequence in public databases. Therefore, the NTD of XynB may contain a new type of CBM. In this experiment, BSA was used as a negative control. 

## 4. Discussion

*Endo*-β-1,4-xylanases (EC 3.2.1.8) play a crucial role in xylan degradation and, thus, have important applications in industries, such as textile, paper, food, animal feed, *etc*. Therefore, searching for novel xylanases with better properties for specific applications from different environments is a long-lasting interest, although xylanases have been extensively studied for many years. In this study, a xylanase gene, *xynB*, was cloned from the marine bacterium, *G.* *mesophila* KMM241, and expressed in *E. coli*. The recombinant XynB is a GH family 8 multi-domain *endo*-β-1,4-xylanase containing an NTD with unknown function and a catalytic domain of GH family 8. Up to now, only four *endo*-β-1,4-xylanases belonging to GH family 8 have been characterized. They are XynY from *Bacillus* sp. KK-1 [7], PhXyl from *P. haloplanktis* [8], XYL6806 from an insect gut microbe [9] and rXyn8 from an environmental DNA library [10]. Among these GH family 8 xylanases, XynB has the highest identity (38%) to the recombinant xylanase rXyn8. In the four GH family 8 xylanases, except XYL6806, which is a modular enzyme containing an NTD with an unknown function, a catalytic domain and a CBM, the other three are mono-domain enzymes containing only a catalytic domain (Figure 1). Blast analysis showed that the NTD of XynB has no obvious homology to any sequence in public databases. Our sequence alignment showed that the homology between the NTD of XynB and that of XYL6806 is only 13.44%. Therefore, XynB is a structurally novel xylanase of GH family 8.

The recombinant XynB has a pH optimum of 6–7 and a temperature optimum of 35 °C. The *K*_m_ and *k*_cat_ value of XynB is 5.82 mg/mL and 609/s, comparative to those of rXyn8 (5.3 mg/mL and 588/s) [10] and lower than that of PhXyl (28 mg/mL and 1247/s) [8]. XynB was thermolabile and salt-tolerant, probably because it was from a marine bacteria isolated from an invertebrate from the Arctic sea [16]. XynB is the first reported salt-tolerant xylanase in GH family 8, which is comparable to some other marine xylanases [14,15,25]. In previous reports, Cu^2+^ seems to strongly inhibit the activity of several xylanases [26,27,28]. Therefore, Cu^2+^ is considered as one of the main adverse factors for industrial application of xylanase [29]. However, our study showed that Cu^2+^ had no evident effect on XynB activity, while Co^2+^, Zn^2+^, Ni^2+^, Mn^2+^ and Li^2+^ had an inhibitory effect. This result is in accordance with the other GH family 10 xylanase, XynA, from *G.* *mesophila* KMM241 [14], but different from PhXyl, which is inhibited by heavy metals, such as Hg^2+^, Cu^2+^, Zn^2+^ and Ni^2+^ [8]. 

Substrate specificity analysis showed that the recombinant XynB was a strict *endo*-xylanase and did not have any β-glucosidase activity, which is in accordance with all the other reported family 8 *endo*-xylanases. However, the hydrolysis product profiles of different family 8 *endo*-xylanases seem to be quite different. XynB seems to function in a similar way to XYL6806 and PhXyl. All of these three enzymes did not hydrolyze xylotriose, and activity on xylotetraose was too low to detect. They hydrolyzed xylopentaose into xylobiose and xylotriose in a low catalytic efficiency, but efficiently hydrolyzed xylohexaose into xylobiose, xylotriose and xylotetraose [8,9]. rXyn8 functions in a different way, selectively releasing xylotriose from substrates [10]. Structural comparison of rXyn8 and PhXyl showed that subtle amino acid changes in the glycon, as well as the aglycon sub-sites probably form the basis of the observed differences between GH family 8 xylanases [6].

Xylanases have potential applications in a wide range of industrial processes, such as food processing, animal feeds, paper and pulp, textiles and bioremediation, *etc*. [2]. As a strict *endo*-xylanase, XynB may have a potential in some of these industrial processes. Especially, XynB is thermolabile, which may have advantages in the food industries, where a high temperature for enzyme inactivation is not allowed. As a salt-tolerant enzyme, xynB may have a potential in biotechnological processes where the catalysis environment is highly salty, such as in the processing of sea food and saline food.

Many glycoside hydrolases, especially those utilizing insoluble matter as substrates, are modular enzymes with two or more modules, including catalytic modules and carbohydrate-binding modules (CBMs). CBMs are now classified into 66 families on the basis of amino acid sequence similarities in the CAZy database. It is generally considered that CBMs facilitate the access of enzymes to polysaccharides and promote polysaccharides degradation [30,31]. Some xylanases own one or several CBMs. For example, a thermostable multi-domain 1,4-β*-*xylanase from *Caldibacillus cellulovorans* has a CBM at its *N*-terminus [32]. A multi-domain *endo*-β-1,4-xylanase from *Paenibacillus curdlanolyticus* B-6 has three family 22 CBMs at its *N*-terminus and one family 9 CBM at its *C*-terminus [33]. Among the characterized family 8 *endo*-xylanases, only XYL6806 is a modular enzyme containing a family 3 CBM at its *C*-terminus [9]. In this study, the NTD of XynB was found to have insoluble polysaccharide-binding ability, indicating that it may contain a CBM. As the amino acid sequence of the NTD of XynB has no obvious homology to any CBM sequence in public databases, XynB may contain a new type of CBM, of which the structure and function need to be further elucidated.

## 5. Conclusion

Gene *xynB* cloned from the marine bacterium, *Glaciecola mesophila* KMM241, encodes a multi-domain xylanase XynB of GH family 8, which has a low identity (38%) to the characterized xylanases. The recombinant XynB with an apparent *M_r_* of about 95 kDa contains an NTD with an unknown function and a catalytic domain. The recombinant XynB showed optimal activity at pH 6–7 and 35 °C. XynB is an *endo*-xylanase hydrolyzing *xylo*-oligosaccharide with at least five sugar moieties into xylotetraose, xylotriose and xylobiose. XynB was thermolabile and salt-tolerant, which may endue it an advantage in some biotechnological processes. The recombinant NTD exhibited high binding ability to insoluble polysaccharides, such as xylan and avicel. The NTD shows no obvious homology to any other CBM sequence in public databases, suggesting that XynB may contain a new type of CBM. These results indicate that XynB is a novel xylanase of GH family 8.

## Supplementary Files

Supplementary File 1 Supplementary Data (PDF, 102 KB)
