# Supplementary material for: Gene Cloning, Expression and Characterization of a Novel Xylanase from the Marine Bacterium, Glaciecola mesophila KMM241
Source: Mar Drugs. 2013 Apr 8;11(4):1173–87. doi: 10.3390/md11041173 (PMC3705397; doi:10.3390/md11041173)

Supplementary Data

Figure S1. Lineweaver-Burk plots curves for the hydrolysis of beech wood xylan (**A**) and oat spelt xylan (**B**). The initial rates were determined with 1.0–15 g/L substrate at 35 ℃. The data represent the mean of three experimental repeats with SD ≤ 5%.

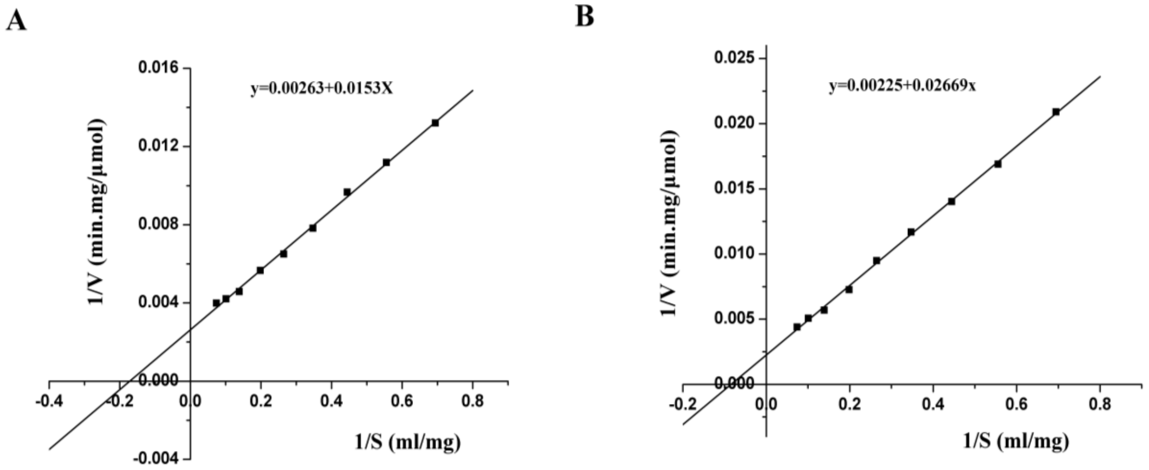

Supplement: Supplementary File 1 — Supplementary Data (PDF, 102 KB) [file marinedrugs-11-01173-s001.pdf]
